# Investigation of *N*-Acetyltransferase 2-Mediated Drug Interactions of Amifampridine: In Vitro and In Vivo Evidence of Drug Interactions with Acetaminophen

**DOI:** 10.3390/pharmaceutics15051471

**Published:** 2023-05-11

**Authors:** Yeo-Dim Park, Yoon-Jee Chae, Han-Joo Maeng

**Affiliations:** 1 College of Pharmacy, Gachon University, Incheon 21936, Republic of Korea; 2 College of Pharmacy and Research Institute of Pharmaceutical Sciences, Woosuk University, Wanju 55338, Republic of Korea

**Keywords:** amifampridine, *N*-acetyltransferase 2, acetaminophen, 3-*N*-acetylamifampridine, drug interactions

## Abstract

Amifampridine is a drug used for the treatment of Lambert–Eaton myasthenic syndrome (LEMS) and was approved by the Food and Drug Administration (FDA) of the United States (US) in 2018. It is mainly metabolized by *N*-acetyltransferase 2 (NAT2); however, investigations of NAT2-mediated drug interactions with amifampridine have rarely been reported. In this study, we investigated the effects of acetaminophen, a NAT2 inhibitor, on the pharmacokinetics of amifampridine using in vitro and in vivo systems. Acetaminophen strongly inhibits the formation of 3-*N*-acetylamifmapridine from amifampridine in the rat liver S9 fraction in a mixed inhibitory manner. When rats were pretreated with acetaminophen (100 mg/kg), the systemic exposure to amifampridine significantly increased and the ratio of the area under the plasma concentration–time curve for 3-*N*-acetylamifampridine to amifampridine (AUC_m_/AUC_p_) decreased, likely due to the inhibition of NAT2 by acetaminophen. The urinary excretion and the amount of amifampridine distributed to the tissues also increased after acetaminophen administration, whereas the renal clearance and tissue partition coefficient (K_p_) values in most tissues remained unchanged. Collectively, co-administration of acetaminophen with amifampridine may lead to relevant drug interactions; thus, care should be taken during co-administration.

## 1. Introduction

**Figure 1:**
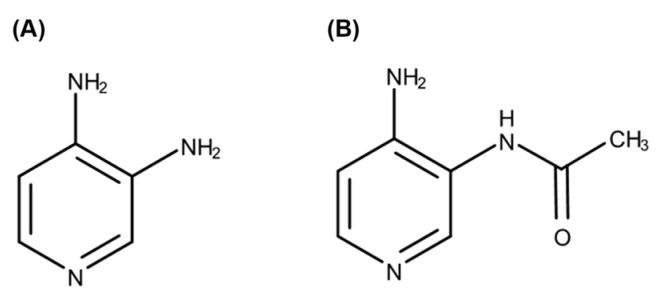
Chemical structures of amifampridine (**A**) and its metabolite, 3-*N*-acetylamifampridine (**B**).

Lambert–Eaton myasthenic syndrome (LEMS) is a rare autoimmune disorder of the neuromuscular junction. In patients with LEMS, antibodies against voltage-gated calcium channels decrease the release of acetylcholine into neuromuscular synapses, resulting in muscle weakness [1]. Amifampridine (Figure 1A), a potassium channel blocker, increases calcium influx, resulting in exocytosis of synaptic vesicles containing acetylcholine [2]. Clinical trials have confirmed that amifampridine effectively controls fatigability and weakness in patients with LEMS, and its adverse effects are generally tolerable, with mild to moderate severity [3]. The Food and Drug Administration (FDA) of the United States (US) approved amifampridine tablets for the treatment of LEMS in adults (Firdapse^®^) as well as in patients 6 to 17 years of age (Ruzurgi^®^) in 2018 [4]. Amifampridine remains the only FDA-approved evidence-based treatment option for LEMS.

In humans, amifampridine is rapidly absorbed after oral administration and reaches its maximum plasma concentration (C_max_) within 1 h, which is delayed by food intake [5]. It is extensively metabolized to 3-*N*-acetylamifampridine (Figure 1B), a pharmacologically inactive metabolite mainly formed by *N*-acetyltransferase 2 (NAT2). NAT1 is also involved in the metabolism of amifampridine to 3-*N*-acetylamifampridine at a much slower rate [6]. NAT2 genes are highly polymorphic in humans, and slow acetylators exhibit significantly higher blood levels of amifampridine, with more frequent adverse events [4,7], suggesting a significant contribution of NAT2 to the pharmacokinetics and safety of amifampridine. More than 90% of the dose of amifampridine (parent drug, 19%) and 3-*N*-acetylamifampridine (metabolite, 74.0–81.7%) is excreted via the urine within 24 h after administration. The elimination half-life is approximately 2.5 h for amifampridine and 4 h for 3-*N*-cetylamifampridine in humans [4]. 

Acetylation is one of the pathways involved in the detoxification and biotransformation of many drugs [8]. NATs are phase II metabolizing enzymes that transfer an acetyl group from acetyl coenzyme A (acetyl-CoA) to a xenobiotic acceptor substrate [9,10] and are responsible for the acetylation of aromatic and heterocyclic amines as well as hydrazines. The mechanism is a ping-pong bi-bi reaction involving two successive steps. First, catalytic Cys68 is acetylated by binding to acetyl-CoA. Acetyl-CoA is then released, followed by substrate binding and transfer of an acetyl group to the exocyclic amine or the oxygen of the hydroxylated amine in the second reaction [11,12]. NAT2 is an enzyme found in many organs, such as the liver, lungs, breast, colon, ureter, and prostate [13], and catalyzes numerous clinically used drugs such as dapsone, procainamide, isoniazid, phenelzine, and sulfonamide [14,15]. Additionally, several NAT2 inhibitors, including acetaminophen, apocynin, and cimetidine, have been reported [16,17,18]. Especially, the inhibitory effects of acetaminophen on NAT2 have been reported both in vitro and in vivo [8,16]. Acetaminophen inhibited sulfamethazine acetylation in human liver cytosol isolated from fast and slow acetylators (*K_i_* value of 2144 and 712 µM in fast and slow acetylators, respectively) and also the acetylation of sulfamethazine decreased in humans when acetaminophen was co-administered. These previous findings indicate that acetylation inhibition by acetaminophen is likely to induce clinically relevant drug interactions. However, drug interaction studies of acetaminophen with drugs mainly metabolized by the *N*-acetylation process, such as amifampridine, are still quite limited. Considering its widespread use and high therapeutic dose (usual dose of 1000–2000 mg/day and maximum recommended therapeutic dose of 4000 mg/day), the potential for drug interactions between acetaminophen and NAT2 substrate drugs should be thoroughly investigated. 

Unexpected alterations in drug pharmacokinetics may lead to decreased efficacy and increased toxicity, eventually resulting in treatment failure. In the case of amifampridine, increased systemic exposure may induce nervous system disorders based on its mechanism of action [19]. However, investigations on NAT2-mediated drug interactions with amifampridine have rarely been reported. Therefore, this study aimed to investigate the effects of NAT2 inhibitors, including acetaminophen, on the pharmacokinetics of amifampridine, a NAT2 substrate, using in vitro and in vivo systems.

## 2. Materials and Methods

### 2.1. Materials

Acetaminophen, acetyl-CoA, amifampridine, apocynin, cimetidine, dimethyl sulfoxide (DMSO), formic acid, phosphate-buffered saline (PBS), Rompun 2% injection, and sodium chloride were purchased from Sigma Aldrich (St Louis, MO, USA). 3-*N*-acetylamifampridine and ondansetron were obtained from Toronto Research Chemicals (North York, ON, Canada) and Cadila (Ahmedabad, India), respectively. Heparin was purchased from Huons (Seongnam, Korea) and Zoletil was obtained from Vibrac SA (Carros, France). High-performance liquid chromatography (HPLC) grade methanol and acetonitrile from Honeywell Burdick and Jackson Co. (Ulsan, Korea) were used in this study. Liver S9 fractions of rats, humans, mice, and rat plasma were purchased from Sekisui XenoTech (Kansas City, KS, USA). Water was purified using an AquaMAX ultrapure water purification system (YL Instruments, Anyang, Korea). All other chemicals and solvents were of reagent or HPLC grade and were used as received without further purification. 

### 2.2. In Vitro Metabolic Profiles of Amifampridine in Rat Liver S9 Fraction

An in vitro metabolic study was performed to investigate the metabolic profile of amifampridine in the rat liver S9 fraction. The reaction mixture, which consisted of rat liver S9 fraction (2 mg/mL protein) and 2 mM acetyl-CoA in potassium phosphate buffer, was prepared and incubated for 5 min in a thermal mixer (200 oscillations/min) at 37 °C. Then, amifampridine was added to the reaction mixture to make the final concentrations of amifampridine at 2, 10, 20, 50, 100, 500, and 1000 μM. After further incubation for 15 min at 37 °C, 50 μL of the sample was transferred into the tube containing 200 μL of ice-cold potassium phosphate buffer and mixed by vortexing. An amount of 50 μL of the mixture was then transferred into the tube containing 100 μL of internal standard (IS; 5 ng/mL ondansetron in acetonitrile), followed by vortexing and centrifugation at 14,000 rpm for 15 min at 4 °C. The supernatant was transferred into vials for HPLC-tandem mass spectrometry (MS/MS) analysis and the concentration of 3-*N*-acetylamifampridine was determined.

### 2.3. In Vitro Inhibition of Amifampridine Acetylation in the Liver and Intestinal S9 Fractions

To determine the half maximal inhibitory concentration (IC_50_) value of NAT2 inhibitors (acetaminophen, apocynin, and cimetidine) on the metabolism of amifampridine to 3-*N*-acetylamifampridine, the reaction mixture consisted of a liver S9 fraction (2 mg/mL protein) and 2 mM acetyl-CoA in potassium phosphate buffer was pre-incubated for 5 min in a thermomixer at 37 °C. Then, the NAT2 inhibitors (final concentrations of 0, 1, 5, 10, 50, 100, 500, 1000, and 5000 μM) and amifampridine (final concentration of 50 μM) were added to the reaction mixture and incubated for 15 min at 37 °C. An amount of 50 μL of the mixture was then transferred into the tube and mixed with 100 μL of IS (5 ng/mL ondansetron in acetonitrile). The mixture was vortexed and centrifuged at 14,000 rpm for 15 min at 4 °C. The supernatant was transferred into vials for HPLC-MS/MS analysis and the concentration of 3-*N*-acetylamifampridine was determined. 

The inhibitory effects of acetaminophen on amifampridine acetylation were compared in rat liver and intestine S9 fractions. The rat S9 fraction was prepared following a method previously reported [20]. The reaction mixture consisted of rat liver or intestinal S9 fraction (2 mg/mL protein), and 2 mM acetyl-CoA in potassium phosphate buffer was pre-incubated for 5 min in a thermomixer at 37 °C. Then, acetaminophen (final concentration of 300 μM) and amifampridine (final concentration of 50 μM) were added to the reaction mixture and incubated for 15 min at 37 °C. The reaction mixture was then pre-treated and analyzed as mentioned above.

### 2.4. Mechanism of Metabolism Inhibition of Amifampridine by Acetaminophen

To determine the inhibition mechanism of amifampridine metabolism by acetaminophen, various concentrations of amifampridine (40, 80, 160, and 320 μM) were incubated with the rat liver S9 fraction in the presence of different concentrations of acetaminophen (0, 10, 50, 100, and 500 μM). After pre-incubating the reaction mixture containing 2 mM acetyl-CoA for 5 min in a thermomixer at 37 °C, acetaminophen and amifampridine were added and further incubated for 15 min at 37 °C. The samples were diluted five-fold using potassium phosphate buffer, then 50 μL of the diluted samples was transferred into the tube containing 100 μL of IS (5 ng/mL ondansetron in acetonitrile). The mixture was vortexed and centrifuged at 14,000 rpm for 15 min at 4 °C. The supernatant was transferred into vials for HPLC-MS/MS analysis and the concentration of 3-*N*-acetylamifampridine was determined.

To examine whether acetaminophen could inhibit the metabolic activity of amifampridine in a time-dependent manner, the experiment was conducted by using a high concentration of amifampridine (approximately four-fold *K_m_* value) for different pre-incubation times (0, 10, 30, and 60 min) in the presence of different concentrations of acetaminophen (0, 10, 50, 100, and 500 μM). The reaction mixture contained the liver S9 fraction (2 mg/mL protein) and 2 mM acetyl-CoA in potassium phosphate buffer. Then, acetaminophen was added and pre-incubated at different times in a thermomixer at 37 °C. The reaction was then initiated by adding amifampridine (240 μM as a final concentration) and vortex mixing followed by incubation at 37 °C for 15 min. After incubation, the 50 μL aliquot was transferred into a tube containing 200 μL of ice-cold potassium phosphate to dilute it five-fold. Then, the 50 μL of the mixture was transferred into the tube containing 100 μL of IS (5 ng/mL ondansetron in acetonitrile) to terminate the reaction, followed by vortexing and centrifugation at 14,000 rpm for 15 min at 4 °C. Finally, the supernatant was transferred to liquid chromatography (LC) vials for analysis, and the concentration of 3-*N*-acetylamifampridine was determined by LC-MS/MS.

### 2.5. In Vivo Pharmacokinetic Interaction Study in Rats

Male Sprague Dawley (SD) rats (7–8 weeks old, 260–330 g) were purchased from Orient Bio, Inc. (Seongnam, Korea). The rats were housed under a light/dark cycle for 12 h and were left for at least a week before the experiment to adapt to the laboratory environment. Water and food were provided ad libitum. The rats were fasted overnight with free access to water prior to pharmacokinetic studies. The protocols for animal experiments were approved in accordance with the Guidelines for Animal Care and Use of Gachon University (approval no.: GIACUC-R2021006; 13 May 2021).

The rats were anesthetized with a mixture of Rompun and Zoletil by intramuscular injection and received an intraperitoneal injection of vehicle (40:60 PEG400:normal saline; *n* = 8) or 100 mg/kg acetaminophen (*n* = 8). After 10 min, 2 mg/kg amifampridine (dissolved in saline) was administered orally. Blood samples were collected at 0, 5, 15, 30, 60, 90, 120, 240, and 360 min and centrifuged at 14,000 rpm for 15 min at 4 °C. For analysis of amifampridine and 3-*N*-acetylamifampridine, 50 μL of plasma samples was mixed with 100 μL IS solution (5 ng/mL ondansetron in acetonitrile) and the mixture was vortexed followed by centrifugation at 14,000 rpm for 15 min at 4 °C. For the analysis of acetaminophen, 200 μL of IS (tolbutamide 200 ng/mL in methanol) was added to the plasma samples which were diluted 10-fold using the blank plasma and then vortexed for one minute. Further, the samples were centrifuged at 14,000 rpm for 15 min at 4 °C. The supernatants were transferred to LC vials for quantification. 

Urine samples were collected at intervals of 0–4, 4–8, and 8–24 h after the administration of amifampridine to control and acetaminophen-treated rats (*n* = 6), as described above, and were diluted 500-fold with potassium phosphate buffer. An amount of 200 μL of IS was then added to the samples and vortexed for a minute. After centrifugation at 14,000 rpm for 15 min at 4 °C, the supernatant was transferred into vials for quantification of amifampridine and 3-*N*-acetylamifampridine. 

To investigate the effects of acetaminophen on the tissue distribution of amifampridine and 3-*N*-acetylamifampridine, rats were sacrificed at 60 min after administration of amifampridine in control and acetaminophen-treated rats (*n* = 6) as mentioned above, and the tissues, including liver, kidney, heart, lungs, spleen, brain, and muscle tissues, were obtained. Tissue samples were homogenized using a Wheaton^TM^ Dounce tissue grinder (Wheaton Millipore, Billerica, MA, USA) after adding a three-fold volume of PBS. An amount of 100 μL of tissue homogenates was transferred to the tube and 200 μL of IS solution was then added and vortexed followed by centrifugation at 14,000 rpm for 15 min at 4 °C. The supernatants were transferred to LC vials for quantification. For muscle samples, the supernatant was concentrated using a desiccator and reconstituted with the mobile phase solvent. 

### 2.6. Quantification of In Vitro and In Vivo Samples Using LC-MS/MS

An Agilent 6490 Triple Quadrupole MS coupled with an Agilent Technologies 1260 HPLC system (LC-MS/MS) was used to quantify amifampridine and 3-*N*-acetylamifampridine. The mass spectrometer was operated in multiple reaction monitoring (MRM) modes with positive electrospray ionization (ESI+). The MRM transitions for amifampridine, 3-*N*-acetylamifampridine, and IS (ondansetron) were *m*/*z* 110.1→93.0, 152.1→110.1, and 294.1→170.0, respectively. The collision energy was 20, 16, and 28 V for amifampridine, 3-*N*-acetylamifampridine, and IS, respectively. Simultaneous chromatographic separation of amifampridine and 3-*N*-acetylamifampridine was performed using a reversed-phase HPLC column (Synergi Polar—RP column 80 Å, 150 × 2.0 mm, 4 µm; Phenomenex (Torrance, CA, USA)) with a linear gradient of 0.1% formic acid in water and acetonitrile (95%:5%→30%:70%) at a flow rate of 0.2 mL/min for 17 min. The column oven was set to 25 °C and the injection volume was 2 µL.

For the analysis of acetaminophen, MRM mode with ESI+ was used with the MRM transitions of *m*/*z* 152.1→110 and 271.1→155 for acetaminophen and IS (tolbutamide), respectively. The collision energy for both acetaminophen and IS was 14 V. The chromatographic separation was performed with Synergi 4 μM and a Polar-RP column 80 Å (150 × 2.0 mm; Phenomenex) with a linear gradient of 0.1% formic acid in water and acetonitrile (80%:20%→20%:80%) at a flow rate of 0.2 mL/min for 20 min. The column oven was set to 25 °C and the injection volume was 2 µL.

Calibration curves were constructed by plotting the peak area ratios of the analytes to IS on the *y*-axis against their concentration on the *x*-axis using weighted (1/x) least-squares linear regression analysis.

### 2.7. Data Analysis

The *V_m_* and *K_m_* values, Michaelis–Menten parameters, were determined by linear regression using Lineweaver–Burk equation, as follows:

$$\frac{1}{v}=\frac{K_{m}}{V_{max}}\times \frac{1}{\left[S\right]}+\frac{1}{V_{max}}$$

where *v* is the rate of amifampridine metabolism to 3-*N*-acetylamifampridine in the S9 fraction, [*S*] is the concentration of amifampridine, *V_max_* is the maximum metabolic rate of amifampridine, and *K_m_* is the concentration of amifampridine at half *V_max_*. The intrinsic clearance (CL_int_) was calculated using *V_max_/K_m_*, with the assumption that *K_m_* is much greater than [*S*], as is usual in clinical practice. 

The inhibition mode was determined using Lineweaver–Burk plots, and *K_i_* was calculated using the following equation, assuming mixed inhibition:

$$v=\frac{V_{max}\times \left[S\right]}{K_{m}\times \left(1+\frac{I}{K_{i}}\right)+\left[S\right]\times \left(1+\frac{I}{\alpha \times K_{i}}\right)}$$

where *v* is the rate of metabolism to 3-*N*-acetylamifampridine at each concentration of amifampridine, *V_max_* is the maximum rate of metabolism, *K_m_* is the Michaelis constant, I is the concentration of acetaminophen, *K_i_* is the inhibition constant, and [*S*] is the amifampridine concentration. The α value is the degree to which the binding of acetaminophen changes the affinity of the enzyme for amifampridine. 

*K_obs_*, the observed rate of enzyme loss, was estimated from the negative slopes of the lines using a linear regression analysis of the natural logarithm of metabolite formation as a function of incubation time [21]. The first-order rate constant, *K_inact_*, which represents the maximum rate of enzyme inactivation, was calculated using a nonlinear regression analysis as follows:

$$K_{obs}=\frac{K_{inact}\times \left[I\right]}{K_{i}+\left[I\right]}$$

Non-compartment analysis (NCA) was performed to calculate the pharmacokinetic parameters for the in vivo study using WinNonlin (Version 8.3. (Pharsight Corporation, Mountain View, CA, USA)). The area under the plasma concentration–time curve from time 0 to infinity (AUC_inf_) was calculated by the linear trapezoidal method and standard extrapolation using λ, where λ represents the slope of the terminal phase in a semi-log graph for plasma concentration–time profiles. Renal clearance (CL_r_) was determined by dividing the accumulated amount of drug excreted in the urine by AUC_inf_. 

A two-tailed unpaired Student’s *t*-test was performed to determine statistically significant differences between groups and *p*-values less than 0.05 were considered significant. Data are presented as means ± SD. 

## 3. Results

### 3.1. In Vitro Metabolic Profiles of Amifampridine in Rat Liver S9 Fractions

**Figure 2:**
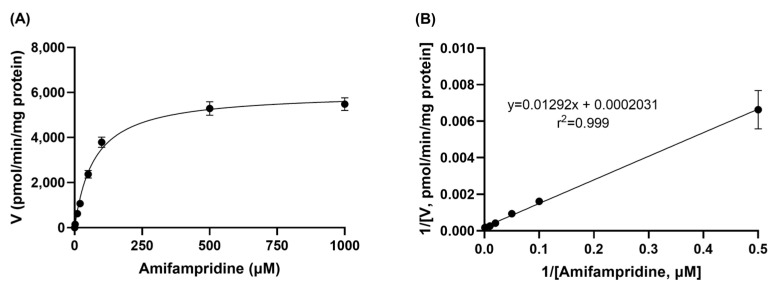
Velocities of 3-*N*-acetylamifampridine formation in rat liver S9 fraction at various concentrations of amifampridine (**A**). The *K_m_* and *V_max_* values were determined using a Lineweaver–Burk plot (**B**). Data are expressed as the means ± SD (*n* = 3).

The formation rate of 3-*N*-acetylamifampridine, a metabolite of amifampridine, in the rat liver S9 fraction was saturated with an increase in the concentration of amifampridine (Figure 2A). Based on the Lineweaver–Burk plot, the *K_m_*, *V_max_*, and CL_int_ values were calculated to be 66.6 ± 24.2 μM, 5130 ± 1100 pmol/min/mg protein, and 0.078 ± 0.011 mL/min/mg protein, respectively, in rat liver S9 fractions (Figure 2B). 

### 3.2. In Vitro Inhibitory Effects of NAT2 Inhibitors on Amifampridine Metabolism

**Figure 3:**
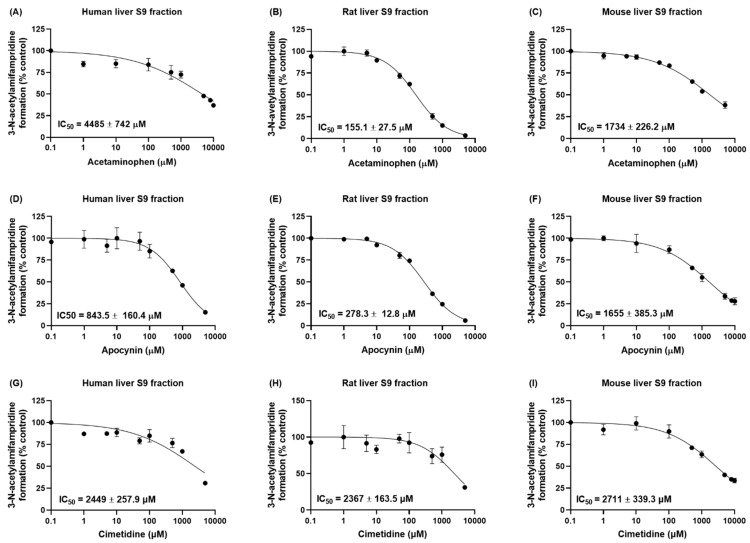
Inhibitory effects of acetaminophen (**A**–**C**), apocynin (**D**–**F**), and cimetidine (**G**–**I**) on the metabolism of amifampridine to 3-*N*-acetylamifampridine in vitro in human, rat, and mouse liver S9 fractions. Data are expressed as the means ± SD (*n* = 3).

The inhibitory effects of NAT2 inhibitors (acetaminophen, apocynin, and cimetidine) on the formation of 3-*N*-acetylamifampridine from amifampridine were determined in vitro using S9 fractions obtained from human, rat, and mouse livers (Figure 3). The effect of acetaminophen on the formation of 3-*N*-acetylamifampridine was more potent in rats than in mice and humans (IC_50_ values of 4485 ± 742 μM, 155.1 ± 27.5 μM, and 1734 ± 226.2 μM for humans, rats, and mice, respectively, Figure 3A–C). The IC_50_ values of apocynin in human, rat, and mouse liver S9 fractions were 843.5 ± 160.4 μM, 278.3 ± 12.8 μM, and 1655 ± 385.3 μM, respectively (Figure 3D–F). The inhibitory potency of cimetidine in rats was similar to that observed in mouse and human S9 fractions (IC_50_ values of 2449 ± 257.9 μM, 2367 ± 163.5 μM, and 2711 ± 339.3 μM in human, rat, and mouse S9 fractions, respectively, Figure 3G–I). All NAT2 inhibitors tested in this study showed inhibitory effects on the metabolism of amifampridine to 3-*N*-acetylamifampridine, suggesting pharmacokinetic interactions between amifampridine and NAT2 inhibitors. 

The inhibitory effects of acetaminophen on amifampridine acetylation were compared in rat liver and intestinal S9 fractions. When 300 µM of acetaminophen was incubated with amifampridine (50 µM) in rat liver or intestinal S9 fractions, the acetylation of amifampridine decreased by 64.7 or 63.3%, respectively (Supplementary Figure S1), suggesting that the inhibitory effects of acetaminophen on amifampridine acetylation in rat liver and intestinal S9 fractions are comparable.

### 3.3. Determination of Inhibition Mode for Acetaminophen on the Metabolism of Amifampridine

**Figure 4:**
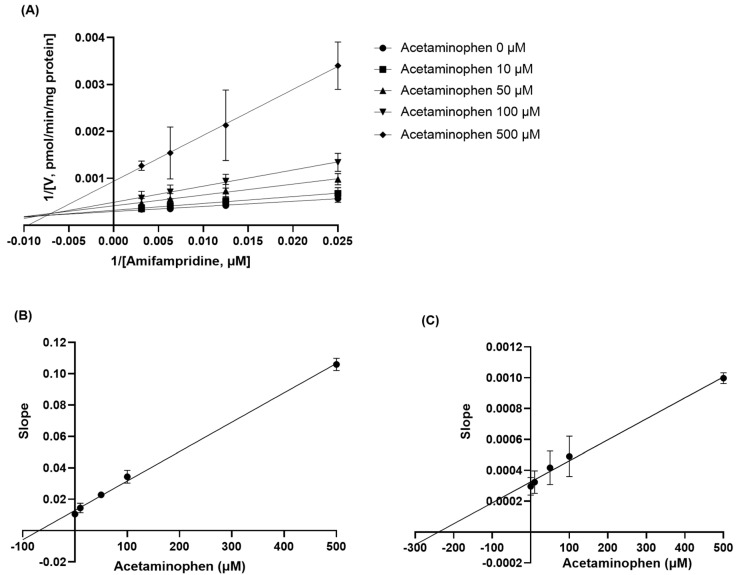
(**A**) Lineweaver–Burk plots of amifampridine metabolism to 3-*N*-acetylamifampridine in rat liver S9 fractions. Amifampridine was incubated in rat liver S9 fractions in the presence of various concentrations of acetaminophen and the amount of formed 3-*N*-acetylamifampridine was measured. (**B**) The secondary plot for *K_i_* was developed using the slope obtained in the Lineweaver–Burk plot versus the concentration of acetaminophen. (**C**) The secondary plot for α*K_i_* was developed using the y-intercept of the linear regression in the Lineweaver–Burk plot versus the concentration of acetaminophen. Data are expressed as means ± SD (*n* = 3).

The reversible inhibition of amifampridine metabolism by acetaminophen was further determined using rat liver S9 fractions treated with various concentrations of amifampridine and acetaminophen. As shown in Figure 4A, acetaminophen inhibited the metabolism of amifampridine in a mixed inhibition manner, increasing the *K_m_* value and decreasing the *V_max_* value. The *K_i_* value calculated from the secondary plot was 68.66 ± 11.85 μM (Figure 4B) and the α value was determined as 3.48, which is not close to 1 (α*K_i_* = 238.8 ± 25.6 μM, Figure 4C), indicating that acetaminophen inhibits the acetylation of amifampridine in a mixed inhibition manner, as observed in the Lineweaver–Burk plot. 

**Figure 5:**
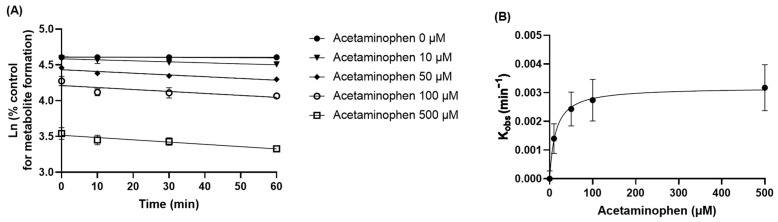
Kinetic profiles of amifampridine metabolism to 3-*N*-acetylamifampridine after pre-incubation with various concentrations of acetaminophen in rat liver S9 fractions. (**A**) A linear regression analysis of the natural logarithm of the percentage of metabolite formation versus pre-incubation time was performed. (**B**) A nonlinear analysis of *K_obs_* versus acetaminophen concentrations was performed to calculate *K_inact_* values (0.0031 min^−1^). Data are expressed as means ± SD (*n* = 3).

The time-dependent inhibition of amifampridine metabolism to 3-*N*-acetylamifampridine by acetaminophen was also evaluated by pre-incubating the liver S9 fraction with acetaminophen (Figure 5). *K_obs_* increased with increasing acetaminophen concentration, reaching saturation at high acetaminophen concentrations. The *K_inact_* value, determined from a nonlinear analysis of *K_obs_* versus acetaminophen concentrations, was 0.0031 min^−1^, indicating that the inhibitory mechanisms of acetaminophen on amifampridine metabolism are time-dependent and that the metabolic activity of amifampridine decreases by approximately 0.31% per min when acetaminophen is pre-incubated with rat liver S9 fractions. 

### 3.4. In Vivo Pharmacokinetic Interaction Study in Rats

**Figure 6:**
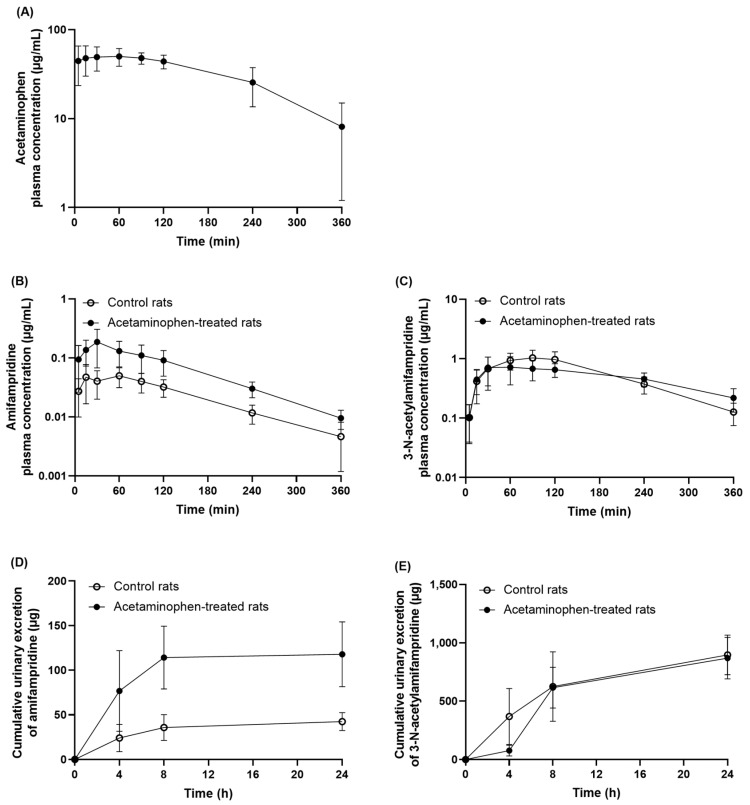
Pharmacokinetic profiles of acetaminophen and amifampridine in rats. (**A**) Acetaminophen was administered intraperitoneally at 100 mg/kg to rats and the plasma concentrations of acetaminophen were quantified (*n* = 6). (**B**,**C**) Amifampridine (2 mg/kg) was orally administered to rats in control (open circles) and acetaminophen-treated rats (closed circles; *n* = 8 in each group) and the plasma concentrations of amifampridine and 3-*N*-acetylamifampridine were determined. (**D**,**E**) Amifampridine (2 mg/kg) was orally administered to rats in control (open circles) and acetaminophen-treated rats (closed circles; *n* = 6 in each group) and the amount of amifampridine and 3-*N*-acetylamifampridine excreted into urine was determined. Data are expressed as means ± SD.

When acetaminophen was intraperitoneally administered into rats at 100 mg/kg, the C_max_ was observed as 54.8 μg/mL (362.5 μM, Figure 6A), which is higher than the IC_50_ value of acetaminophen observed in the in vitro study with rat liver S9 fractions, suggesting the potential interactions of acetaminophen with amifampridine in vivo. Therefore, a pharmacokinetic study was performed in rats to investigate the in vivo pharmacokinetic interactions between acetaminophen and amifampridine. When acetaminophen (100 mg/kg) was administered to rats 10 min prior to oral administration of amifampridine (2 mg/kg), the systemic exposure to amifampridine significantly increased (208.34% and 185.64% increase in C_max_ and AUC_inf_, respectively, Table 1 and Figure 6B). In the case of 3-*N*-acetylamifampridine, the C_max_ decreased slightly after pre-treatment with acetaminophen (27.11% decrease, *p* < 0.05), and the AUC_inf_ was not significantly altered (Table 2 and Figure 6C). However, the AUC_m_/AUC_p_ ratio significantly decreased from 24.48 ± 3.10 to 8.63 ± 2.65 (*p <* 0.001) after pre-treatment of acetaminophen, indicating that acetaminophen markedly inhibits the metabolism of amifampridine to 3-*N*-acetylamifampridine in vivo in rats. 

In addition, urine samples were collected for 24 h to investigate the effects of acetaminophen on the urinary excretion of amifampridine and 3-*N*-acetylamifampridine in rats. As shown in Figure 6D and 6E, the urinary excretion of amifampridine increased at each time point after pre-treatment with acetaminophen and the total urinary recovery of amifampridine was 6.7% and 18.9% (*p* < 0.001) in the control and acetaminophen-treated rats, respectively. The amount of 3-*N*-acetylamifampridine excreted into the urine for up to 4 h decreased from 49.9% to 12.2% after pre-treatment with acetaminophen (*p* < 0.05), whereas the total amount of 3-*N*-acetylamifampridine excreted via urine for up to 24 h was not significantly different between the groups. The renal CL (CL_r_) remained unchanged for both amifampridine and 3-*N*-acetylamifampridine (Table 1), which supports that the urinary excretion pathway of amifampridine is unlikely to be affected by acetaminophen treatment in rats.

**Figure 7:**
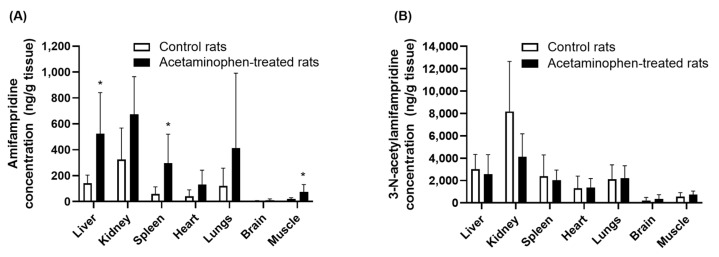
Tissue concentrations of amifampridine (**A**) and 3-*N*-acetylamifampridine (**B**) at 60 min after oral administration of amifampridine (2 mg/kg) in control (open bar, *n* = 5) and acetaminophen-treated rats (100 mg/kg; closed bar, *n* = 6). Data are expressed as means ± SD. * denotes *p* < 0.05, compared to the control group.

The effect of acetaminophen on the tissue distribution of amifampridine and 3-*N*-acetylamifampridine was investigated by calculating the tissue partition coefficient (K_p_: the ratio calculated by dividing the concentration in the tissue by that in the plasma) after oral administration of 2 mg/kg amifampridine to the control and acetaminophen-treated rats. The concentrations of amifampridine in each tissue and the K_p_ of amifampridine and 3-*N*-amifampridine in the liver, kidney, spleen, heart, lungs, brain, and muscles at 60 min are presented in Figure 7 and Table 2. The concentrations of amifampridine in the liver, spleen, and muscle were significantly increased by 270.26%, 265.17%, and 413.11% (*p* < 0.05), respectively, in acetaminophen-treated rats compared with control rats. The kidneys, heart, lungs, and brain of the acetaminophen-treated group also showed a slight increase in amifampridine concentration, although the change was not statistically significant. The calculated K_p_ of amifampridine in the kidneys of acetaminophen-treated rats decreased by 69.43% compared to that in control rats (Figure 7A). However, there were no significant differences in the K_p_ values in other tissues between the control and acetaminophen-treated rats (Table 2). In contrast to amifampridine, no significant difference was observed between the control and acetaminophen-treated rats in the concentration of 3-*N*-acetylamifampridine in the tissues (Figure 7B). The calculated K_p_ of 3-*N*-acetylamifampridine in the kidney in the acetaminophen-treated rats was decreased compared to control rats (16.6 ± 12.4 and 5.1 ± 1.6 in control and acetaminophen-treated rats, respectively), but the difference was not statistically significant. In other organs, the calculated K_p_ values were similar between the groups (Table 2). 

## 4. Discussion

Amifampridine is primarily eliminated via metabolism to 3-*N*-acetylamifampridine, which is formed mainly by NAT2 and NAT1, and the NAT1 has a much slower metabolic rate than NAT2 [5]. The C_max_ and AUC of amifampridine are approximately 3.5-fold and 5–10-fold higher in slow acetylators than in fast acetylators for NAT2 [19]. In addition, a more than 10-fold higher frequency of drug-related treatment-emergent adverse events has been reported with slow acetylators than with fast acetylators for NAT2 [19]. Thus, NAT2 activity is critical for maintaining treatment efficacy with minimal adverse effects. However, to the best of our knowledge, information regarding the drug interactions of amifampridine mediated by NAT2 has not yet been reported, which possibly limits the effective treatment of LEMS with amifampridine. Therefore, we investigated the effects of NAT2-mediated interactions with amifampridine using in vitro and in vivo systems. 

In this study, we confirmed that amifampridine was metabolized through the acetylation pathway in the rat liver S9 fraction. We then evaluated the in vitro inhibitory effects of the well-known NAT2 inhibitors acetaminophen [16], apocynin [17], and cimetidine [18,22] in liver S9 fractions obtained from humans, rats, and mice (Figure 3). The IC_50_ value of cimetidine on NAT2 was calculated as 2367 µM in rat liver S9 fraction, which is similar to the value in a previous report (IC_50_ = 2060 µM [22]). In contrast, the IC_50_ value of apocynin on NAT2 obtained in this study (278.3 µM in rat liver S9 fractions) was less than the previous report (690 µM [17]). However, considering the dependency of IC_50_ values on experimental conditions, such as substrate or enzyme concentrations, the IC_50_ value obtained in this study seems within a reasonable range. The inhibitory effects of cimetidine on the acetylation of amifampridine were similar in human, rat, and mouse liver S9 fractions; acetaminophen and apocynin showed substantial species differences in their inhibitory effects. In particular, an approximately 29-fold stronger inhibitory effect of acetaminophen was observed in rats compared to that in humans (IC_50_ values of 155.1 μM and 4485 μM in rat and human liver S9 fractions, respectively). These results indicate that meticulous care is required in the interpretation of NAT2 inhibitory study results obtained in species other than humans. 

To further investigate the inhibitory mechanisms of acetaminophen on *N*-acetylation of amifampridine, rat liver S9 fraction was treated with various concentrations of amifampridine and acetaminophen and the formation rate of 3-*N*-acetylamifampridine was determined (Figure 4). We observed that acetaminophen inhibits *N*-acetylation of amifampridine in a mixed inhibition manner, which implies that acetaminophen can bind not only with unbound NAT2 but also bound NAT2 with amifampridine, resulting in altered binding affinities of substrates to the enzyme and a maximum metabolism capacity. Acetaminophen also inhibited the *N*-acetylation of amifampridine in a time-dependent manner, with a *K_inact_* of 0.0031 min^−1^ (Figure 5), indicating that the metabolic activity of NAT2 on amifampridine decreased by approximately 0.31% per minute in the presence of acetaminophen. The underlying mechanisms of the acetaminophen-mediated inhibition of amifampridine acetylation determined in this study will help understand the interactions between acetaminophen and amifampridine.

Since acetaminophen strongly inhibited the metabolism of amifampridine in the rat liver and intestinal S9 fractions in vitro, the potential of in vivo pharmacokinetic interactions between amifampridine and acetaminophen was investigated in rats (Figure 6). The dose of amifampridine (2 mg/kg) was determined based on the clinical dose and several pharmacokinetic studies of amifampridine in rats [4,23]. The dose of acetaminophen (100 mg/kg) was selected based on clinical doses and pharmacokinetic interaction studies with acetaminophen in rats [24,25]. The systemic exposure to amifampridine was significantly increased and the total clearance (CL/F) decreased when acetaminophen was administered to rats. However, t_1/2_ remained unchanged after treatment with acetaminophen, likely due to both a decreased CL/F and volume of distribution (V_d_/F) (Table 1), which affect t_1/2_. The reasons behind a decreased V_d_/F remain unclear; however, the increased F, possibly due to inhibition of first-pass intestinal and hepatic metabolism of amifampridine by acetaminophen before reaching systemic circulation, and the decreased distribution of amifampridine into several tissues by acetaminophen may be considered as the relevant mechanisms. Similarly, the increased F can affect the CL/F of amifampridine in the presence of acetaminophen. To confirm the effect of acetaminophen on the systemic CL and V_d_ of amifampridine, an additional in vivo PK study with intravenous administration of amifampridine would be necessary. The AUC ratio of 3-*N*-acetylamifampridine to amifampridine (AUC_m_/AUC_p_) significantly decreased by 65% after pretreatment with acetaminophen (Table 1), suggesting that the pharmacokinetic profiles of amifampridine and its metabolite were strongly affected by acetaminophen in vivo. Furthermore, a highly significant increase was observed in the cumulative urinary recovery of amifampridine in acetaminophen-treated rats compared to control rats. The renal clearance of amifampridine did not differ between the groups, which may be due to the proportional increase in cumulative urinary recovery to the increase in AUC_inf_ in the plasma of acetaminophen-treated rats. In addition, the accumulated amount excreted in the urine or renal clearance of 3-*N*-acetylamifamrpdine was not altered by treatment with acetaminophen. Taken together, the increased systemic exposure to amifampridine and the decreased AUC_m_/AUC_p_ are unlikely to be attributable to the interaction with acetaminophen via the urinary excretion pathway of amifampridine or its metabolite. 

A tissue distribution analysis showed that the amount of amifampridine distributed in the tissue was higher in acetaminophen-treated rats than in control rats (Figure 7). However, the K_p_ values, which were calculated by dividing the concentration of amifampridine in the tissue by that in the plasma, were not significantly altered by treatment with acetaminophen, except in the kidneys (Table 2). The K_p_ value of 3-*N*-acetylamifampridile was not significantly altered by acetaminophen in any tissue studied. These results suggested that the mechanisms governing the tissue distribution of amifampridine and 3-*N*-acetylamifampridile were not significantly associated with acetaminophen, except in the kidneys. The K_p_ value of amifampridine in the kidneys decreased by 51% in acetaminophen-treated rats. This was likely a result of the increased urinary excretion of amifampridine in acetaminophen-treated rats. The mechanism underlying this finding remains unclear, and further studies are necessary to elucidate it. However, the altered distribution of amifampridine only to the kidneys after treatment with acetaminophen is less likely to affect the plasma concentrations of amifampridine, considering the volume of the kidney and total plasma volume. Collectively, the inhibitory effects of acetaminophen on the *N*-acetylation of amifampridine seem to be the most plausible mechanism to explain the in vivo pharmacokinetic interactions between acetaminophen and amifampridine, based on the in vitro and in vivo results (urinary excretion and tissue distribution studies). 

It is generally known that acetaminophen is safe and less likely to induce clinically important drug interactions; thus, this drug is available over the counter as well as by prescription in most countries. However, several recent studies have provided evidence that acetaminophen may induce drug interactions by altering the pharmacokinetics of victim drugs. For example, systemic exposure to sorafenib, a tyrosine kinase inhibitor used for the treatment of advanced renal cell carcinoma, and its metabolite N-oxide sorafenib increased in the presence of acetaminophen in rats [26], which may be due to P-glycoprotein (P-gp) inhibition by acetaminophen [27]. In addition, acetaminophen increases the clearance of lamotrigine glucuronide conjugates, resulting in decreased systemic exposure to lamotrigine in humans [28]. Our study also highlights the importance of acetaminophen-mediated drug interactions; thus, drug interactions with acetaminophen should receive more attention regarding their clinical implications. However, in our study, acetaminophen inhibited the amifampridine metabolism much more strongly in the rat liver S9 fraction than in the human liver S9 fraction, and species differences should be carefully considered when interpreting the in vivo interaction results in rats and humans. The observed C_max_ values after oral or intravenous administration of acetaminophen are ~200 µM at most in humans [29,30]. Considering the IC_50_ of acetaminophen on NAT2 inhibition in human liver S9 fractions and the pharmacokinetic profiles of acetaminophen reported in humans, the potency of drug interactions might be lower than those observed in rats. Further translational research between different species and clinical studies are warranted to obtain more confirmative evidence on the drug interactions between amifampridine and acetaminophen in humans. Since the NAT2 gene is expressed in the tissues other than the liver, such as the intestine [31], the tissue distribution of the NAT2 gene in different species should be also considered. 

This study was originally designed to investigate the systemic drug interactions between acetaminophen and amifampridine via hepatic NATs, and thus acetaminophen was administered by the intraperitoneal route. However, acetaminophen administered via the intraperitoneal route was absorbed rapidly and possibly affected the intestinal and hepatic first-pass metabolism of amifampridine, which was administered orally. Additionally, acetaminophen distributed into the tissues is likely to affect metabolism of amifampridine during systemic circulation not only in the liver but also in the intestine. Thus, diverse processes may be involved in the observed in vivo interactions between amifampridine and acetaminophen in rats, even though the level of contribution in each process cannot be separated quantitatively from this study. We confirmed that the inhibitory effects of acetaminophen on amifampridine acetylation are comparable between rat liver and intestinal S9 fractions; however, more comprehensive and systematic information on the interaction between amifampridine and acetaminophen, including a comparison of the inhibitory effects of acetaminophen in human and rat intestinal S9 fractions, is required to facilitate translation of the interaction effect into humans.

To the best of our knowledge, this is the first study to investigate the NAT2-mediated drug interaction potential of amifampridine with acetaminophen in vitro and in vivo. This study reported that acetaminophen inhibited the metabolism of amifampridine, leading to altered pharmacokinetics in rats. Therefore, care should be taken when amifampridine is co-administered with acetaminophen. 

## 5. Conclusions

This study investigated the interactions between amifampridine and acetaminophen using in vitro and in vivo systems. An inhibitory effect of acetaminophen on the acetylation of amifampridine was observed in vitro and the in vivo pharmacokinetic profile of amifampridine was affected by acetaminophen treatment in rats. In addition, the ratio of systemic exposure of 3-*N*-acetylamifampridine to amifampridine decreased after treatment with acetaminophen. Acetaminophen increased the urinary excretion and the amount of amifampridine distributed to the tissues. Acetaminophen co-administration may lead to relevant drug interactions with amifampridine; thus, care should be taken when co-administering amifampridine and acetaminophen.

## Figures and Tables

**Table 1:** Pharmacokinetic parameters of amifampridine and 3-*N*-acetylamifampridine after oral administration of amifampridine (2 mg/kg) in control and acetaminophen-treated rats (100 mg/kg; *n* = 8 in each group). Data are expressed as means ± SD.

	Amifampridine		3-*N*-acetylamifampridine	
Parameter	Control Group	Acetaminophen- Treated Group	Control Group	Acetaminophen-Treated Group
C_max_ (μg/mL)	0.07 ± 0.02	0.20 ± 0.10 **	1.21 ± 0.24	0.88 ± 0.26 *
T_max_ (min)	46.87 ± 36.25	60 ± 35.86	78.75 ± 31.82	90.00 ± 68.03
AUC_last_ (μg∙min/mL)	8.47 ± 1.4	24.85 ± 7.13 ***	204.82 ± 39.12	180.56 ± 20.29
AUC_inf_ (μg∙min/mL)	9.11 ± 1.61	26.03 ± 6.99 ***	221.42 ± 40.74	221.33 ± 23.47
t_1/2_ (min)	83.92 ± 21.12	81.34 ± 24.98	84.94 ± 21.61	142.78 ± 54.62 *
MRT (min)	137.65 ± 42.85	124.94 ± 31.64	159.05 ± 33.42	230.16 ± 77.95 *
CL/F (mL/min/kg)	225.39 ± 38.29	81.74 ± 21.23 ***	-	-
V_d_/F (mL/kg)	26,913.30 ± 6740.87	9830.78 ± 4689.82 ***	-	-
CL_r_ (mL/min)	4.89 ± 1.71	3.87 ± 0.91	2.94 ± 1.55	3.58 ± 0.6
AUC_m_/AUC_p_	-	-	24.48 ± 3.10	8.63 ± 2.65 ***

AUC_inf_, area under the plasma concentration–time curve from time 0 to infinity; AUC_last_, area under the plasma concentration-time curve from time zero to the last quantifiable point; AUC_m_/AUC_p_, AUC ratio of 3-*N*-acetylamifampridine to amifampridine; CL/F, clearance divided by bioavailability (F); CL_r_, renal clearance; C_max_, maximum plasma concentration; T_max_, time to reach C_max_; MRT, mean residence time; t_1/2_, half-life; V_d_/F, volume of distribution divided by F. *, **, and *** denote *p* < 0.05, *p* < 0.01, and *p* < 0.001, respectively, compared to the control group.

**Table 2:** Tissue partition coefficient (K_p_) of amifampridine and 3-*N*-acetylamifampridine after oral administration of amifampridine (2 mg/kg) at 60 min in control and acetaminophen-treated rats (100 mg/kg). Data are expressed as means ± SD.

	Amifampridine		3-*N*-amifampridine	
Tissue	Control (*n* = 5)	Acetaminophen (*n* = 6)	Control (*n* = 5)	Acetaminophen (*n* = 6)
Liver	3.8 ± 2.6	3.0 ± 1.5	6.5 ± 5.0	3.0 ± 0.8
Kidney	7.4 ± 2.7	3.6 ± 0.4 *	16.6 ± 12.4	5.1 ± 1.6
Spleen	1.0 ± 0.5	1.6 ± 1.0	3.3 ± 0.7	3.0 ± 0.9
Heart	0.6 ± 0.5	0.7 ± 0.5	1.8 ± 0.5	1.6 ± 0.4
Lungs	1.7 ± 1.1	2.2 ± 2.8	2.9 ± 0.5	2.7 ± 0.9
Brain	0.1 ± 0.3	0.1 ± 0.0	0.2 ± 0.2	0.2 ± 0.2
Muscle	0.5 ± 0.4	0.4 ± 0.2	0.9 ± 0.2	0.9 ± 0.3

K_p_ values were calculated by dividing the concentration of amifampridine or 3-*N*-amifampridine in the tissue by that in the plasma. * *p* < 0.05, compared with control rats.

## Data Availability

All data are included in this manuscript.

## Supplementary Materials

The following supporting information can be downloaded at: https://www.mdpi.com/article/10.3390/pharmaceutics15051471/s1, Figure S1: Inhibitory effects of acetaminophen (300 µM) on the metabolism of amifampridine (50 µM) to 3-*N*-acetylamifampridine in vitro in rat liver and intestinal S9 fractions. Data are expressed as the mean ± SD (*n* = 3).
