# Supplementary material for: Investigation of N-Acetyltransferase 2-Mediated Drug Interactions of Amifampridine: In Vitro and In Vivo Evidence of Drug Interactions with Acetaminophen
Source: Pharmaceutics. 2023 May 11;15(5):1471. doi: 10.3390/pharmaceutics15051471 (PMC10221159; doi:10.3390/pharmaceutics15051471)

SUPPLEMENTAL INFORMATION

Investigation of *N*-Acetyltransferase 2-Mediated Drug Interactions of Amifampridine: In Vitro and In Vivo Evidence of Drug Interactions with Acetaminophen

Yeo-Dim Park, Yoon-Jee Chae and Han-Joo Maeng

Supplementary Figure S1.

Inhibitory effects of acetaminophen (300 μM) on the metabolism of amifampridine (50 μM) to 3-N-acetylamifampridine in vitro in rat liver and intestinal S9 fractions. Data are expressed as the mean ± SD (n=3).

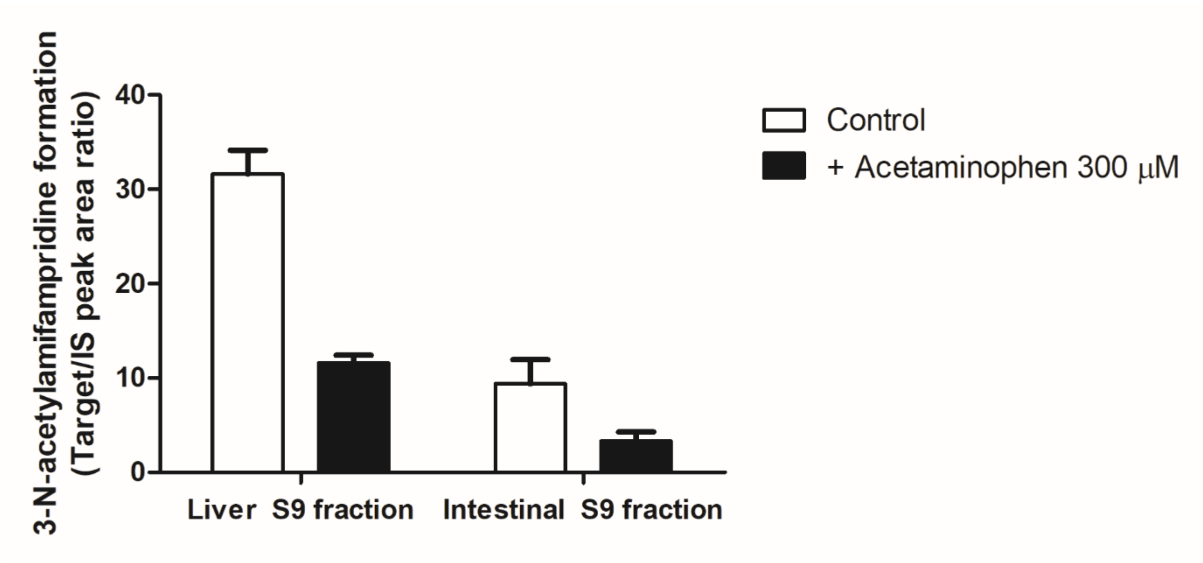

Supplement: Supplementary file 1 [file pharmaceutics-15-01471-s001.zip › pharmaceutics-2278426-supplementary.pdf]
